# ESMira: A decentralized open-source application for collecting experience sampling data

**DOI:** 10.3758/s13428-023-02194-2

**Published:** 2023-08-21

**Authors:** David Lewetz, Stefan Stieger

**Affiliations:** Department of Psychology and Psychodynamics, Karl Landsteiner University of Health Sciences, Dr. Karl-Dorrek-Straße 30, A-3500 Krems an der Donau, Austria

**Keywords:** Ecological momentary assessment, Open-source, Decentralized, Android, iOS, Smartphones, Ambulatory assessment

## Abstract

This paper introduces ESMira, a server and mobile app (Android, iOS) developed for research projects using experience sampling method (ESM) designs. ESMira offers a very simple setup process and ease of use, while being free, decentralized, and open-source (source code is available on GitHub). The ongoing development of ESMira started in early 2019, with a focus on scientific requirements (e.g., informed consent, ethical considerations), data security (e.g., encryption), and data anonymity (e.g., completely anonymous data workflow). ESMira sets itself apart from other platforms by both being free of charge and providing study administrators with full control over study data without the need for specific technological skills (e.g., programming). This means that study administrators can have ESMira running on their own webspace without needing much technical knowledge, allowing them to remain independent from any third-party service. Furthermore, ESMira offers an extensive list of features (e.g., an anonymous built-in chat to contact participants; a reward system that allows participant incentivization without breaching anonymity; live graphical feedback for participants) and can deal with complex study designs (e.g., nested time-based sampling). In this paper, we illustrate the basic structure of ESMira, explain how to set up a new server and create studies, and introduce the platform’s basic functionalities.

**Supplementary information:**

The online version contains supplementary material available at 10.3758/s13428-023-02194-2.

The design of a study goes hand in hand with the practical question of what data collection methods should be used. Paper-and-pencil questionnaires work very well for cross-sectional studies, but may be less than optimal for longitudinal designs, especially with multiple assessments in a field setting (i.e., in the individual surroundings of participants’ everyday lives; Stone et al., 2002). This problem is even more pronounced for designs with very short intervals (down to several minutes) between data collection time points—so-called experience sampling methods (ESM; Csikszentmihalyi et al., 1977; Csikszentmihalyi & Larson, 1987; Larson & Csikszentmihalyi, 1983, 2014; Mehl et al., 2012; Myin-Germeys & Kuppens, 2022). ESM is known under a variety of names, including ecological momentary assessment (Smyth & Stone, 2003; Stone & Shiffman, 1994), ambulatory assessment (Fahrenberg et al., 2007; Trull & Ebner-Priemer, 2013), or diary methods (Bolger et al., 2003; Ellis-Davies et al., 2012), all of which are based on the idea of “assess[ing] data in *natural settings*, in *real-time* (or close to real-time occurrence) and on *repeated time* occasions” (Conner et al., 2009). Typically, ESM is used as a form of self-report measure where participants are asked to report either at random times (with the help of some kind of notification system) or at fixed intervals, or when a specific event has taken place (i.e., event-based sampling; Bolger & Laurenceau, 2013). This has the advantage of gathering data in the field instead of the laboratory, enabling higher ecological validity, allowing for more diverse responses, and increasing the accuracy of responses through live rather than retrospective reports (Schwarz, 2012; Solhan et al., 2009).

One method for realizing ESM study designs that has been experiencing rapid growth in recent years involves the use of smartphones (Dufau et al., 2011; Harari et al., 2015, 2016; Miller, 2012) and has several advantages over traditional paper-and-pencil methods. Because of their small size, smartphones are easy to carry around and are already familiar to and owned by most people.^1^ With an internet connection, questionnaires can also be transmitted easily and almost instantly, while the time of responses to a questionnaire can be easily traced thanks to digital timestamps automatically obtained from the smartphone’s clock. Smartphones also allow the use of videos, pictures, or interactive items, and, using built-in sensors (e.g., GPS positioning, phone orientation, or recordings via camera), they can add even more (indirect) data (see also ”digital phenotyping”; Insel, 2017). Another big advantage of smartphones is their ability to have timed (or random) notifications, which can be used as pings to fill out questionnaires on a regular basis. “Pings” are notifications triggered by apps, such as notifications from social media platforms to alert users to new messages, and this functionality is also used in most smartphone-based ESM applications.

However, collecting data with smartphones requires software. Smartphone apps need to be programmed, which is often the greatest burden preventing scientists from conducting ESM studies online (especially in the social sciences; for a similar discussion, see Miller, 2012; Piwek & Ellis, 2016). Several platforms are already in place for ESM studies on smartphones (for a list of examples, see Myin-Germeys & Kuppens, 2022; Piwek & Ellis, 2016); however, each has its own challenges.

## Existing ESM platforms for smartphones

Myin-Germeys and Kuppens (2022) list five widely used platforms: ExpiWell (*ExpiWell*, 2018), m-PATH (Mestdagh et al., 2022), movisensXS (*MovisensXS*, 2022), RADAR-base (Ranjan et al., 2019), and SEMA3 (Koval et al., 2019). This list is not exhaustive (a more extensive albeit older list can be found in Piwek & Ellis, 2016) but represents widely used options available today. All five platforms can be used for free to some extent, but m-Path, movisensXS, and ExpiWell require payment after a certain number of participants and for additional features, which seems to be common practice in commercially funded ESM platforms. ESM platforms typically consist of a mobile app installed on participants’ smartphones which can be used to fill out questionnaires, as well as a server that provides study information (i.e., questionnaires, pings, reminders) and collects and stores questionnaire data. The server generally has an admin panel where studies can be created and from which study data can be downloaded (for a more detailed description, see Myin-Germeys & Kuppens, 2022). Usually, study administrators have no influence over which server is used for their study, nor any control over where data are sent or stored. Very few platforms offer the option to use another server than the one available by default (from the previously listed platforms, only RADAR-base has this option). Unfortunately, this process can be cumbersome: first, the setup itself often requires a significant amount of technical expertise; second, in most of the platforms offering this option, a default server address is hard-coded into the app. In other words, if study administrators set up their own server, they also need to publish their own version of the mobile app to make it work with their server. This requires additional technical knowledge and extra costs in order to make the app available in app stores.

In summary, for most platforms, data from participants and study settings are mostly centralized on one server, which is controlled by a single entity. This is called centralization (for an early definition of centralization in computing, see King, 1983), and, apart from other challenges (discussed in Bodó et al., 2021), centralization requires study administrators to entrust their data to a third party and to be dependent on the capabilities of the server that is provided for them.

## Another option: ESMira

ESMira, the platform we present in this paper, combines several features of existing platforms but sets itself apart by being decentralized (i.e., studies and data can be stored on different servers which function independently of each other), easy to set up without much technical knowledge required, and open-source and free to use (its source code is already available on GitHub^2^). ESMira was developed with the requirements of scientific ESM studies in mind (e.g., data security, anonymity, ethical considerations) and can also be used in applied research (e.g., clinical contexts; although ESMira is not yet certified for clinical intervention studies; see “Limitations”).

The major difference from other platforms is its decentralized structure: there is no central server where all data are stored. Instead, ESMira is structured to enable study administrators to set up their own server with very little technical knowledge required. This way, study administrators can ensure that no data are shared with any third party and have full control over where data are sent and stored.

Furthermore, ESMira offers an extensive list of features, including an anonymous built-in chat to contact participants, a reward system that allows for incentivizing participants without breaching their anonymity, and personal statistics for participants. ESMira is also able to deal with complex study designs (e.g., nested time-based sampling).

## How ESMira works

**Fig. 1:**
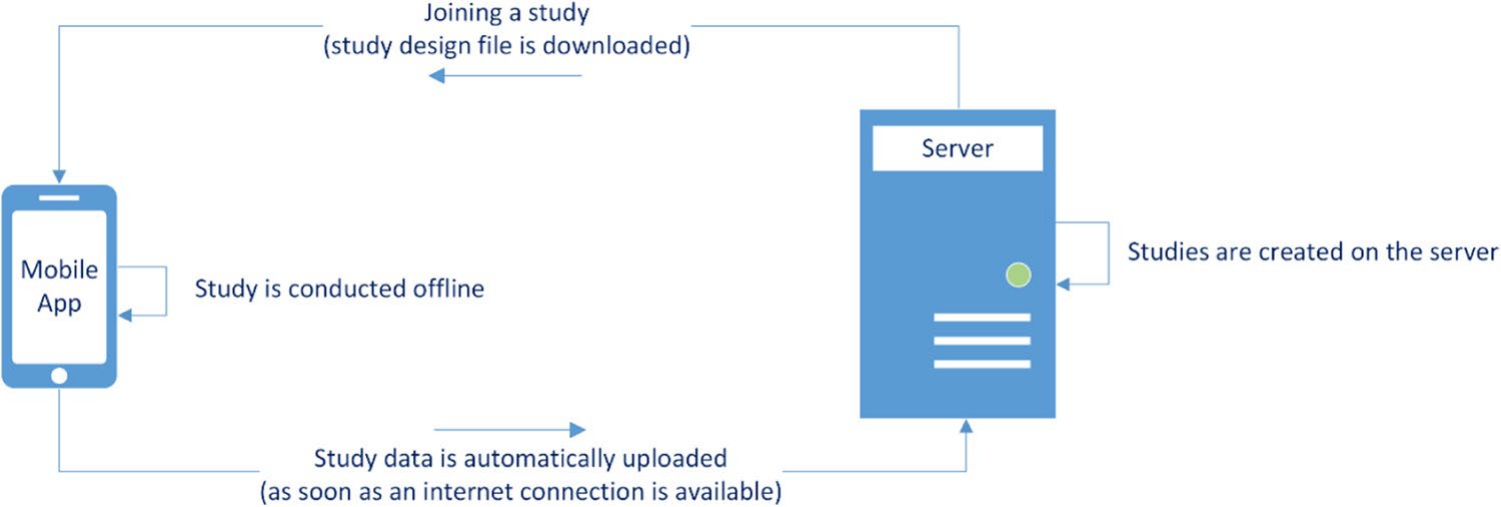
General working model of ESMira

As with other platforms, ESMira consists of two modules (illustrated in Fig. 1): a server where study administrators can set up studies and communicate with participants, and where all study data are stored; and a mobile app (Google’s Android, Apple’s iOS, and a web client) that is used by participants (e.g., when filling out questionnaires).

### The server

The server stores study data and communicates with the mobile app (Android, iOS). The mobile app requires an internet connection to access the server at least twice: once to download the study design file, and at least once after the study has finished to upload all gathered data. Uploading of data occurs automatically once an internet connection is established (Fig. 1). Beyond these occasions, no internet connection is required to participate in a study. The server’s admin panel is also where study administrators can create and manage studies and communicate with participants. Access to the admin panel can be restricted to studies and functionalities (e.g., access to data, changing the study design, access to chat messages; for more information, see section “User and permission management” below).

**Fig. 2:**
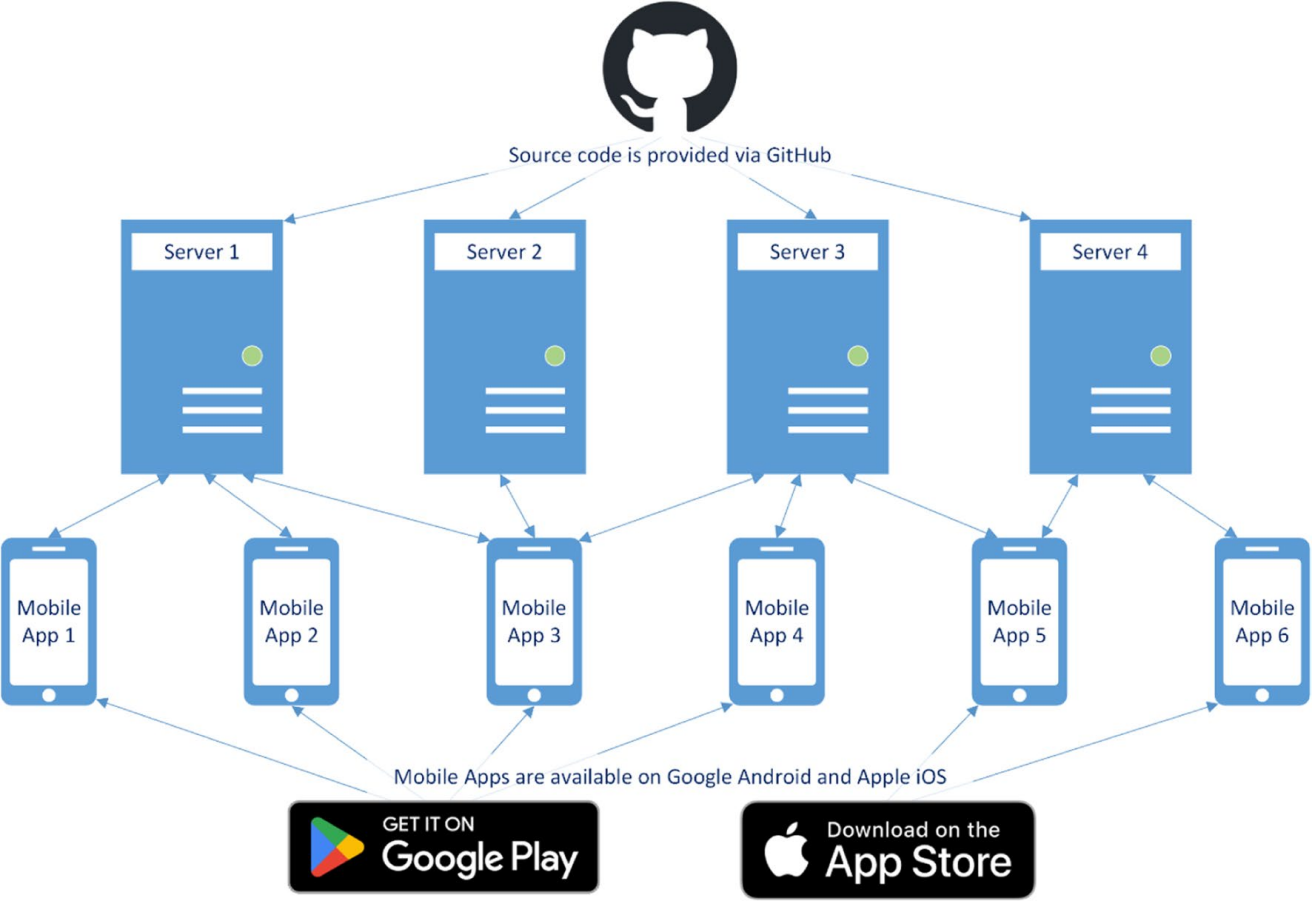
Example of the communication hierarchy of servers and mobile apps. *Note*. ESMira servers are set up using the source code stored on GitHub. Server updates are also installed from GitHub. Mobile apps only communicate with a server if they joined a study on that server. They can join multiple studies on different servers at the same time

As previously mentioned, study administrators are encouraged to set up their own personal server, which is designed to be as straightforward as possible. The server software is programmed using PHP (at least version 7.4), a scripting language that is well known, widely used, available on all common platforms (i.e., operating systems), and already installed on most servers.^3^ It also uses no other dependencies (e.g., database software), meaning that setting up an ESMira server only requires copying the server files onto a web space (for more information, see the section “Setting up an ESMira server” below). Because the server is not dependent on an external database, data can be moved or copied to another location or server without further setup. Each server works independently of the others. When joining a study via the mobile app, all internet communication happens exclusively with the respective server hosting the study. This setup has three major advantages. First, study administrators can ensure that participant data will never be sent to anyone else. Second, because the mobile app is not bound to any specific server, participants can use the official mobile app from the app store, and study administrators are not required to maintain their own version. Third, although unlikely to happen in practice, participants could join multiple studies on different servers at the same time (Fig. 2).

### The mobile app

**Fig. 3:**
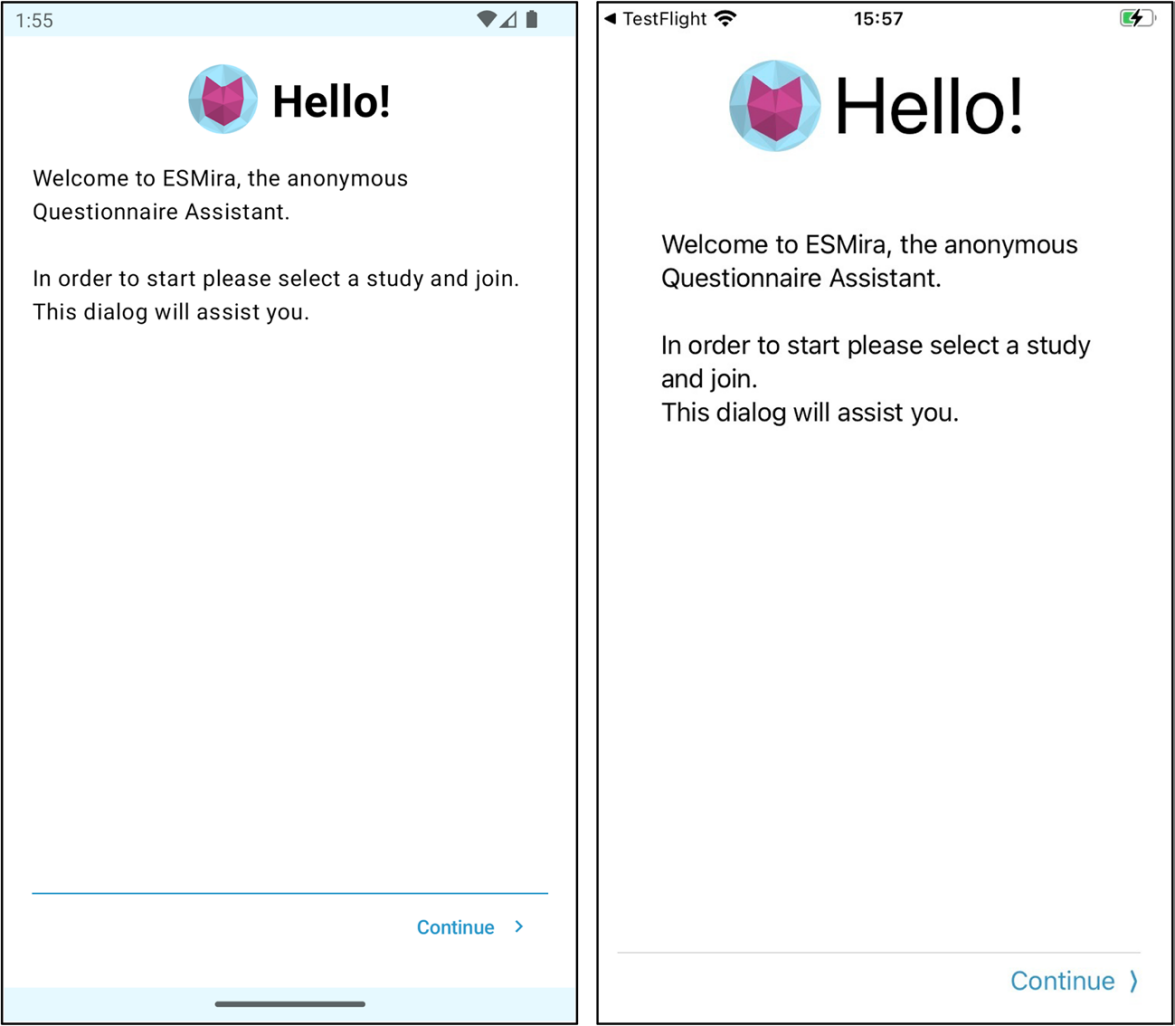
Screenshots of the Welcome screen of the mobile apps on Android (left) and iOS (right)

The mobile app (for example screenshots see Figs. 3 and 6) is used by participants to fill out questionnaires or send anonymous chat messages to the study administrator (or to the user having permission to read and write messages on the server; for more details, see below in “User and permission management”). The mobile app is available on both the Google Play Store (i.e., Android) and Apple App Store (i.e., iOS); both versions are developed natively for their respective platforms, such that they are programmed specifically for one platform and can take full advantage of all device features. This usually provides better performance and better access to hardware- or platform-dependent features such as sensors. It is also possible to use the server’s web interface to fill out questionnaires, so that questionnaires are presented as classical online questionnaires in the web browser; however, to make use of additional features (e.g., pings; personal statistics; reward codes), participants require the ESMira mobile app. The mobile app is not bound to a specific server. To join a study, participants can use a QR code that is automatically generated by the ESMira server and includes the respective server address, select the server from a list, or provide the server URL manually. After installation of the mobile app, ESMira generates a random user ID. This user ID is used for all communications between the mobile app and the server (e.g., study data; messages) to ensure that all data sent to the server are completely anonymous.

## Setting up an ESMira server

**Fig. 4:**
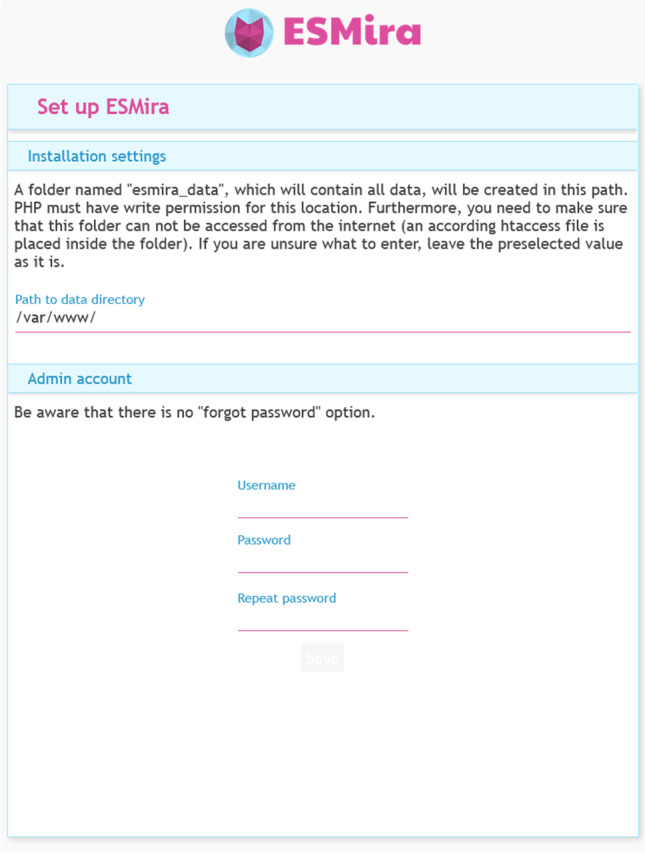
Initial setup of ESMira

Depending on server access and technical knowledge, the initial setup is the only step that might require IT support unless a server space with PHP is already available, as is often the case for university staff. First, the zipped release of ESMira^4^ must be downloaded and unzipped. The unzipped files then need to be transferred to the web space. Permission for the files needs to be set in such a way that PHP has write permissions, usually done by changing the owner of the files to “www-data.” Then, the server address (e.g., https://example.com, https://subdomain.example.com or https://example.com/subfolder) needs to be opened in a browser to choose an admin username and password (see Fig. 4). After that, the server should be ready. More detailed instructions can be found in the section “Setting up a server” of our Wiki.^5^

## Setting up a study

**Fig. 5:**
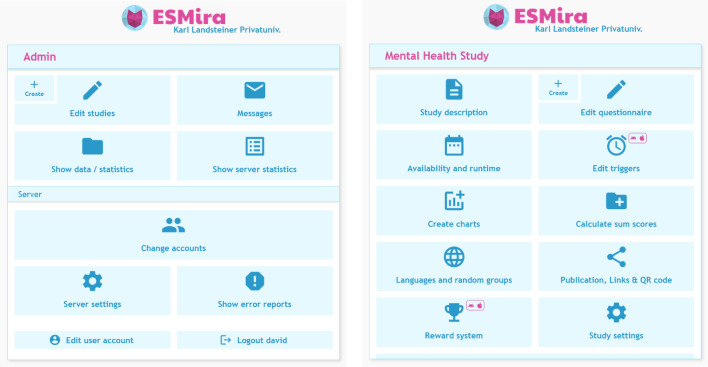
Initial screen of the admin interface (left) and screen of the study overview of an example study (right)

New studies can be created using the ESMira admin interface, which can be opened by adding “?admin” to the server address (e.g., https://example.com/?admin) available by pressing the “Create” button near the “Edit studies” menu button (see Fig. 5). Most texts can be formatted via a “What You See Is What You Get” (WYSIWYG) text editor. A study can have several questionnaires available depending on date, time of day, group, platform, or specific conditions. The system’s flexibility allows for a variety of study designs, including end-of-day questionnaires, event-based sampling, time-based sampling, or one-time questionnaires (e.g., final questionnaire or demographics). Each questionnaire can consist of several pages, and its items can be randomized independently if needed. Several different items can be used in a questionnaire (e.g., lists, text, number or binary input, date and time input, Likert or visual analogue scales, videos, images, photos, or dynamic items; more information can be found in our Wiki^6^).

## Main server features

In addition to developing features for ESMira, we constantly keep security and anonymity in mind and are focused on improving usability and the optical presentation of both the app and the server. Below is a list of the main features we deem especially noteworthy and believe set ESMira apart from similar platforms. The first section is focused on features that are mainly connected to the server. The second section lists features mainly connected to the mobile app.

### Easy server setup

Technological knowledge and programming skills are a hurdle for some study administrators, especially in the social sciences, which can prevent them from using computer-based methods (Yarkoni, 2012). Therefore, since its conception, the installation of the ESMira server was designed to be as easy as possible (see “Setting up an ESMira server”).

### Update the server with just one click

ESMira is continually updated with new features and bugfixes and can do so automatically (Figure S1). When selected (user with admin rights assumed), ESMira will back itself up, download its newest version from GitHub,^2^ and, if needed, update the data structure to the newest version.

### Translated into multiple languages

At the time of writing, ESMira has been translated into English, German, and Ukrainian. We are actively seeking translations into as many languages as possible. For this purpose, we have set up an openly available translation platform,^7^ where others can add new translations or improve existing translations.

### User and permission management

The server provides a sophisticated user management system to fine-tune access to different studies. Different users can have selected access permissions to selected studies, such as to change projects, read and write chat messages, or access data (see Figure S2). For example, a common setup could be that only the project investigator has permission to change the project and access data and messages, while the statistician only has permission to access the data, and the study worker only has permission to access messages to participants (i.e., helpdesk). This allows for clear project administration, with clear permissions regarding who can see which participant’s data.

### Online data viewer

ESMira offers a built-in data viewer, although all study data can also be downloaded as CSV files. The viewer can handle large data sets and also provides a range of useful filters and full-text search options (see Figures S3 and S4) to analyze study data in real time (e.g., how many participants have joined the study; how many participants are currently active within the study).

### Live study statistics and charts

ESMira offers several options to obtain a quick overview of the current state of a study. The general overview shows several statistics about participation (e.g., total quit or joined events) and completed questionnaires over the last days (see Figure S5). Specific statistics show detailed charts for individual participants’ responses (see Figure S6), and for more in-depth analysis, custom charts can give an overview of specific cases.

### Server statistics

ESMira automatically generates overall performance statistics for the whole server, such as the total number of currently active studies, completed questionnaires, participants, devices used, and last active studies (see Figure S7). This is especially useful for ESMira admins to get a quick overview and to identify problems early (e.g., sudden drop in completed questionnaires per day).

### Automatically generated study information page with participation instructions

The web interface provides an automatically generated information webpage on the server for each study. This webpage includes easy-to-follow instructions for potential participants on how to install the mobile app and participate in the study, including a QR code or links to the study (see Figure S8). The webpage link can be sent to participants instantly for recruitment purposes and to simplify enrollment.

### A web interface for questionnaires

If enabled, questionnaires can also be filled out using a web browser (see Figure S9). The web version is useful for questionnaires that do not rely on pings or other app features. It is compatible with most major browsers (e.g., Chrome, Edge, Firefox, Safari, Opera) on desktop or mobile and includes a fallback version that works on older platforms or browsers with JavaScript disabled. Upon first use, participants need to self-select an anonymous user ID, which is then stored in a browser cookie so that participants do not have to enter their user ID in subsequent questionnaires.

### Multiple studies with access keys

Studies can either be publicly available on the server or hidden with a password (“access key”). A study can have multiple access keys, and multiple studies can share the same access key. The access key (if enabled) needs to be provided to participants when enrolling in a study and is also presented on the study information webpage. This procedure is useful to prevent participants from enrolling in the wrong study (if more than one study runs in parallel on the specific ESMira server) or when the study is intended for a closed pool of participants (e.g., workers of a specific company). Furthermore, it not only enables study administrators to track different cohorts in a study by providing participants with different access keys (access keys used by participants can be tracked in the data file) but also allows participants to have access to thematically linked studies with just one access key.

### Different language versions for studies

Sometimes multiple language translations are required for a study (i.e., cross-cultural research). ESMira is designed to handle this (see Figures S10 and S11). During enrollment in a study, the app automatically detects the language a participant’s operating system uses and presents the study in the same language. If the needed language is not available, it will be presented instead in a predefined default language. ESMira can also automatically detect language changes and updates its language used accordingly. In the data files, ESMira logs the language a participant was using (e.g., Figure S4). Furthermore, when designing a study on the ESMira server, each text input option is accompanied by a language selection menu, which can be used to switch the language without having to reload the page (see Figure S10). Therefore, it is not necessary to create a new study for each language version because the language can be changed within the project (e.g., item text).

### Random study groups

ESMira can randomly assign participants to groups for the purposes of a classical between-subject design (Figure S11). Groups can have their own questionnaires and/or interventions, such as group-specific texts, pictures, and videos. This allows for the implementation of psychological experiments or ecological momentary interventions (Heron & Smyth, 2010; Stieger & Lewetz, 2018) when using ESM designs.

### Live updating of studies

Using ESMira, studies can be updated even after a study has started. In general, an update to a study will only affect new participants so as to prevent confusion or intervention effects. But, if necessary, changes can also be released to existing participants (see Figure S12). The mobile app automatically checks approximately every 12 hours for updates to joined studies and automatically downloads them in the background. This is represented in the study version, which is assigned to each data upload to facilitate differentiation between study versions when analyzing the data version (there is a major version number for changes for all participants and minor version number for changes only for new participants; for an example, see Figure S4). The option to update ESMira is especially useful if problems occur during the study, or if time points of time-based sampling procedures are not known at the start of the study (e.g., lineup in sport events; Götz et al., 2020; Stieger et al., 2015).

### Calculation of sum scores into own variables

ESMira can automatically calculate sum scores from other variables (see Figure S13), which can be helpful when generating charts based on scores of specific scales.

### Flexible study design file

All instructional data for a study (e.g., questionnaire items; sampling time points; informed consent; information texts; procedure details such as experimental groups or duration of the study) are saved in a single text file (i.e., popular JSON format) that can be easily edited, exported, or copied (see Figure S14). This allows for editing the study directly in the text file (e.g., adding whole sections to questionnaires by using copy-and-paste) and also enables researchers to publish the complete study design together with the data (i.e., open data, open material). Furthermore, it also makes it possible to create similar studies quickly by duplicating an existing study design (i.e., exact replication using the same design file), including on a different ESMira server.

## Main features of the mobile app

**Fig. 6:**
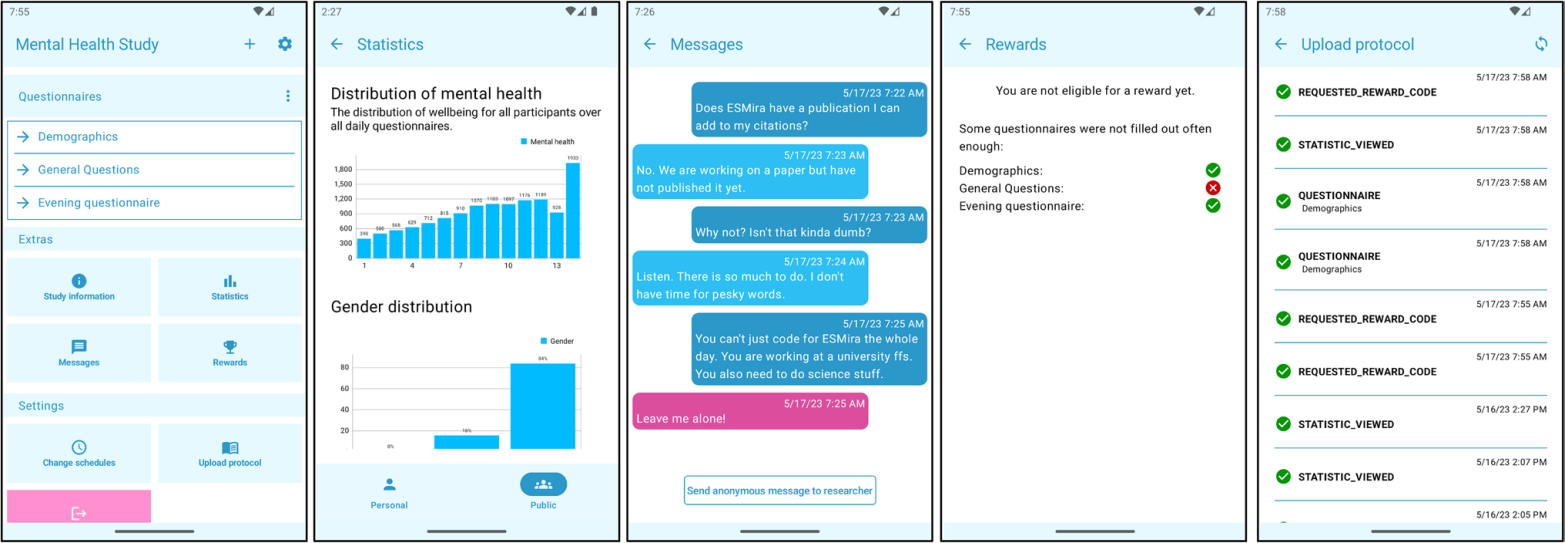
Examples of main screens of the mobile app (in order: Home Screen, Statistics, Messages, Rewards, and Upload Protocol)

The following features are mainly connected to the mobile app. Figure 6 gives examples of the main screens of the mobile app.

### Completely anonymous

When first opening the app, each participant is assigned their own randomly generated user ID (i.e., 12 numbers/letters, e.g., 4mZm-F8vN-5Vyk), used for uploading data or sending messages. Participants do not have to provide any personal information to use the app. Neither the server nor the app uses any external services, and data are sent directly (and only) to the server hosting the study over a secured connection. 8

### Little effort required for participants to join studies

To join a study, participants only need to install the mobile app, use the built-in QR code scanner (see Figure S15) to scan the study’s specific QR code (which is automatically created by the ESMira server), and accept the terms of the study (i.e., informed consent, app permissions). Alternatively, it is also possible to use a URL that automatically opens the study within the mobile app (after the mobile app is installed). Participants can also open the mobile app and enter the study information (server URL and access key) to manually enroll in a study.

### Informed consent and check for permissions

Permissions in ESMira are dealt with in a clear, transparent, and open fashion and are only requested when needed. Before participants can join a study, they get a list of permissions required for a specific study, which they can accept one by one (e.g., notifications, app usage; see Figure S16). The list also includes short explanations for each permission about why it is necessary on the “What for?” information screen. Participants must also agree to an informed consent which is provided by the study administrator.

### Does not need a stable internet connection during a study

When joining a study (which needs an internet connection), all study instructions are downloaded into the app; from this point forward, almost everything happens locally on the participant’s smartphone. When filling out a questionnaire, the response data are first saved inside the app before ESMira tries to automatically upload it to the server as soon as an internet connection is available. Thanks to this system, the internet is only required again to transfer study data to the server, which can happen after the study has already been completed.

### Automatic pings

ESMira supports two different triggers for pings: time-based triggers or app-event-based triggers (see Figure S17). *Time-based triggers* usually occur several times a day (and/or only on specific days of the week/month). For each time-based trigger, multiple times of day can be selected; these, in turn, can either be fixed or chosen randomly within a set time span. *App-event-based triggers* react to specific in-app events (e.g., joining a study, filling out a specific questionnaire, or viewing statistics for a study). Triggers will either elicit a notification on the smartphone that leads directly to a specific questionnaire (i.e., an invitation to a questionnaire), send an automated chat message to the participant, or create a plain notification on the smartphone with custom text.

Furthermore, ESMira offers the possibility of “nested time-based sampling triggers.” For example, if a participant reacts to a random time-based trigger by completing a questionnaire, this event can trigger another time-based trigger, sending a notification to complete another questionnaire within the following 30 minutes. This is especially interesting for intervention studies that analyze the longevity of possible intervention effects (e.g., Stieger et al., 2023).

### Anonymous chat messages between study administrator and participants

ESMira incorporates a chat message system with which participants can communicate with the study administrators anonymously (see Figure S18). Additionally, study administrators can send group messages either to all participants or to only a subgroup of participants selected through various filters (e.g., phone type or app version). To avoid dependency on third-party services, ESMira does not use push notifications (which require a single fixed server registered to Google or Apple services, meaning all messages would have to be sent through our servers and also use an external service provided by Google or Apple), but the app automatically checks for new messages approximately every 12 hours or when the app is started. Thanks to this system, server messages do not rely on a third-party server to be transmitted to participants, but it also means that messages to participants are not transmitted in real time. To account for that, the ESMira server shows if and when a message has been collected by the participant.

### Live graphical feedback

ESMira can automatically generate live graphical feedback, such as personal or general statistics, for participants (as used in Stieger et al., 2021; Stieger, Graf et al., 2022b; Swami et al., 2022). There are several kinds of charts available (e.g., line charts, bar charts, or scatterplots with regression lines; see Figure S19). Each chart has many customization options (e.g., showing frequencies, means, or changes over time) and can either be completely personalized with participants’ data or depict aggregated data from all participants. Additionally, specific data categories can be filtered out using conditions, and charts can be made available only after the study has ended to prevent intervention or reactivity effects (see Eisele et al., 2023). Also, to prevent concerns about anonymity, all personal graphs are generated offline on the participant’s smartphone and are never transferred to the ESMira server.

### Reward code system

Sometimes study administrators incentivize participation in their studies by offering financial rewards, vouchers, or course credit. This can be problematic because participants would need to disclose their user ID to prove that they are eligible to receive an incentive, which breaks anonymity. Therefore, a reward code system was developed to ensure that the anonymity of participants is not violated. Study administrators can define criteria such as participation time (i.e., reward code is only visible to participants after a certain number of days), or a required minimum number of responses to questionnaires (see Figure S20). The mobile app verifies whether these conditions are met. If not, the app shows a message about the missing requirements (e.g., demographic questionnaire missing). If all criteria are met by the participant, an anonymous reward code, which is independent of the user ID, is shown, together with further instructions (see Figure S21). Participants are instructed to send this code to the study administrator, preferably via email or social media, rather than via the in-built chat function (which would leak their user ID). The study administrator can also verify the validity of a reward code on the ESMira server (see Figure S20). This has two advantages: first, the reward code is not connected to the user ID (because both are never saved or sent together), thus enabling participants to disclose any personal information needed to complete payment without being connected to their study data (i.e., staying anonymous); second, it enables the study administrator to check anonymously whether a reward code is valid, to avoid potential abuse.

### Enabling participants to change ping schedules

Participants’ daily schedules can vary drastically (e.g., night shift workers vs. day shift workers), and it is often very difficult to reflect that in a study design. ESMira can give participants the option to change the time schedule of pings by setting either a timeframe for random pings or exact times for fixed pings (see Figure S22).

### Tracking app usage or screen time

If enabled, ESMira is able to track the daily usage time and daily usage count of other apps or the smartphone in general (i.e., screen time) on Android smartphones; unfortunately, such a feature is currently not available on iOS. The feature to track usage time is implemented as a questionnaire item, which shows the data gathered by ESMira (i.e., the respective app, daily usage count, and daily usage time; see Figure S23) of the last full day (i.e., yesterday). This allows participants to verify beforehand what information is sent via ESMira, following best practices in scientific ESM research (see Mehl et al., 2012; Myin-Germeys & Kuppens, 2022).

## Development

The development of ESMira started in early 2019 and is still ongoing. ESMira has been actively used in several studies at Karl Landsteiner University (KLU) of Health Sciences (Stieger, Aichinger, et al., 2022a; Stieger et al., 2020, 2021, 2023; Stieger, Graf, et al., 2022b; Swami et al., 2022; Volsa et al., 2022) and other universities (Pail, 2023; Pöcksteiner, 2022) using different languages (e.g., English, German, Ukrainian) and in different nations (e.g., Canada, USA, Austria, Germany, Ukraine). Currently, we are aware of four further instances of ESMira, in addition to KLU. From early on, the focus of ESMira has been on easy deployment, with very few requirements for study administrators and low effort for participants. It has been tested on various Android smartphones (e.g., HTC, Huawei, LG, Motorola, Samsung, Sony), iOS devices, and browsers (e.g., Chrome, Edge, Firefox, Safari, Opera) on both desktop and mobile devices. Another important factor in the development of ESMira is the focus on participant compliance by being open and clear about data collection and following best practices in scientific ESM research (see Mehl et al., 2012; Myin-Germeys & Kuppens, 2022), as well as providing anonymity and strong data protection.

ESMira was mainly developed for scientific research, without corporate interests in mind. Our decision to be open-source and offer ESMira to the scientific community enables us to seek new collaborations and connect to other researchers in a way that would otherwise not be possible. This will hopefully help to ensure the quality and usability of ESMira. An extensive Wiki is available on GitHub,^9^ with a discussion forum^10^ to ask questions or provide feedback, which we welcome. The source code of ESMira is maintained under the open-source license aGPL,^11^ which is openly available on GitHub.^2^ Others are welcome to audit the code and participate in its development.

## Limitations

Even though we believe that smartphones have great potential in aiding ESM studies, there are limitations and new problems (contrary to classical methods like pen-and-paper) that need to be considered. First, because the behavior, design, and permissions of operating systems on smartphones (e.g., Android and iOS) are constantly changing, ESM apps need to be adapted on a regular basis to ensure their reliability (for a non-app, web-based solution, see Arslan et al., 2020). We actively use ESMira in our own studies, and constantly publish fixes and updates that others can benefit from. Also, because ESMira is open-source, others can add their improvements or bug-fixes themselves. However, new updates can also lead to problems if not considered carefully. Changes can lead to incompatibilities between the smartphone app and the server or can change intended behaviors within ongoing studies. ESMira has a complex version-correction system to ensure that outdated servers or outdated apps can still function properly or display a warning if version differences cannot be resolved automatically, and an update is needed. Even study instruction files are automatically revised before being interpreted to ensure that they still behave the same. Nevertheless, there is still the possibility that some updates can impact ongoing studies, either by unforeseen errors or by changes that interfere with the current study design. We are aware of this possibility and, in the long term, plan to implement a means of informing study administrators directly if critical updates are in the works. In the meantime, major changes are always published first on GitHub, where study administrators can stay informed or ask questions to help them prepare accordingly, and a discussion board is available.^2,8^ Non-critical updates are always published as a test version first and are only made public after sufficient time and testing have passed.

Second, because of the variety of smartphone models and participants’ levels of experience with smartphones, every study usually needs some level of technical support (e.g., solving permission issues with some participants, solving problems with very specific smartphone models, figuring out and correcting various “user errors” or forwarding feedback about app errors that can only be fixed by the app developer). To assist with communication, ESMira has a built-in chat system where participants can ask questions anonymously without much effort and study administrators can contact participants directly if needed. Also, ESMira automatically detects whether major errors or crashes have occurred and then asks the participant to send an error report to the app developer.^12^

Third, the varying levels of sensor accuracy between smartphone models can be problematic, and evidence for most smartphone models is still scarce (some evidence regarding the accuracy of the accelerometer of different devices can be found in Bittel et al., 2016; Grouios et al., 2022; Kuhlmann et al., 2021; regarding the GPS sensor see, Merry & Bettinger, 2019; von Watzdorf & Michahelles, 2010). For example, there are a variety of different Android smartphone models, with varying hardware specifications. Some manufacturers also add their own changes to the Android operating system, which can cause problems. Notifications in particular have been known to fail on some specific brands (e.g., Huawei, Samsung, OnePlus). Several projects have been created to overcome this issue (e.g., dontkillmyapp^13^), but because changes are different for every manufacturer and they are all implemented outside the scope of normal app permissions, solutions depend on the exact smartphone model and on participants manually changing very specific settings on their phone. ESMira can overcome this problem by automatically detecting when notifications were not issued properly and displaying a dialog accordingly. This dialog includes the most common fixes (e.g., sound settings, notification settings, and even loads device specific instructions from dontkillmyapp^13^; see Figure S24). However, participants with less knowledge of smartphones may require additional guidance via the ESMira chat message system. One way of combating issues around different smartphones is to provide participants with separate smartphones instead of having them install ESMira on their personal smartphones. The limitation of this approach is that participants would have to carry an additional device that needs to be regularly charged. However, it ensures that sensors and functionality of all smartphones used in the study can be tested thoroughly and that they behave the same way (or similarly) across participants.

Fourth, while we believe that ESMira is quite mature, there are some functionalities that we deem important but that are not yet available with ESMira. Sensors or devices (e.g., smartwatches, wearables) are typically connected to smartphones via Bluetooth Low Energy (BLE). ESMira is currently not compatible with external sensors or devices (e.g., Internet of Things [IoT] devices). This functionality could be useful for health applications that would benefit from heart rate or any other health-related indicators (see the vast applications from the sports and health sector, e.g., fitness trackers). Furthermore, while ESMira has a basic implementation of sum scores, and the real-time charts have many ways of representing data (see “Live study statistics and charts”), complex real-time calculations to transform participants’ data are not possible yet. We are planning to expand these functionalities in the future, but until then, most data transformations require post-processing using a statistics software (e.g., R, SPSS). Moreover, the options for dynamically changing questionnaires are still limited in ESMira. One advantage of presenting questionnaires in digital form is that questionnaires do not need to be static. Questionnaires could, for example, be adapted to the input of a participant, or display only a sub-selection of items on a randomized basis. ESMira is able to randomize the order of items in questionnaires and can also hide questionnaires according to the participant’s operating system or study group. But apart from that, ESMira currently lacks more sophisticated features, which we hope to be able to address soon. For more details about future developments, see the “Future Directions” subsection in the online supplement.

Fifth, ESMira is not yet certified for clinical interventions (e.g., Medical Device Directive^14^). This might be possible in the future, but ESMira is still in development, and certification would be premature.

## Conclusion

The landscape for smartphone-based tools in ESM research is dynamic. Most existing platforms are costly or require too much technical knowledge to set up, which can act as barriers to study administrators with little resources. Almost all projects focus on a centralized approach, meaning that data are saved on a single server controlled by one entity. ESMira seeks to change this by being freely available and open-source,^2^ as well as making it easy for study administrators to set up their own server (i.e., not centralizing all studies on one server).^3^ Study design files can be easily downloaded in plain text to support open-science practices (e.g., open materials, replications). Participants can use ESMira on Android, iOS, or a web browser. ESMira has a large repertoire of features and has been tested extensively in several projects so far. It is being actively used for scientific research at different universities (e.g., KLU and others), and its development is still ongoing. By making it available to the scientific community, we hope to advance the quality of the ESMira platform, extend its functionality with the help and input of other study administrators, and help facilitate the adoption of ESM research.

### Supplementary Information

ESM 1 (DOCX 2.03 MB)

## Data Availability

Not applicable.

## Footnotes

1 https://www.statista.com/statistics/330695/number-of-smartphone-users-worldwide.

2 https://github.com/KL-Psychological-Methodology/ESMira.

3 https://w3techs.com/technologies/overview/programming_language.

4 https://github.com/KL-Psychological-Methodology/ESMira-web/releases/latest.

5 https://github.com/KL-Psychological-Methodology/ESMira/wiki/Setting-up-a-server.

6 https://github.com/KL-Psychological-Methodology/ESMira/wiki/Setting-up-studies.

7 https://translate.jodli.dev.

8 There are two necessary exceptions in the mobile app: When participants send an error report, this report is sent to our server instead. Furthermore, on Android, when ESMira detects a problem with notifications, it shows a dialog that links to several Android settings. Since some of these settings need to be specific to the user’s smartphone model, and keeping track of such information is very time-consuming, we are using an external service and load some of the instructions from https://dontkillmyapp.com.

9 https://github.com/KL-Psychological-Methodology/ESMira/wiki.

10 https://github.com/KL-Psychological-Methodology/ESMira/discussions.

11 https://www.gnu.org/licenses/agpl-3.0.en.html.

12 Because these kinds of errors involve programming of the app and not the study itself, error reports are not sent to the study administrator but to our server instead. This is the only time where data are sent to a server that is not hosting the study. Although participants can view the contents of the error report beforehand and no study data or personal data are transferred this way, information in the error report can be used to get details about the study itself and the participant’s device.

13 https://dontkillmyapp.com/.

14 https://en.wikipedia.org/wiki/Medical_Devices_Directive.

## Code availability

The source code of the presented applications (server, Android app, iOS app) is available online at GitHub: https://github.com/KL-Psychological-Methodology/ESMira.
