# Supplementary material for: ESMira: A decentralized open-source application for collecting experience sampling data
Source: Behav Res Methods. 2023 Aug 21;56(5):4421–34. doi: 10.3758/s13428-023-02194-2 (PMC11288990; doi:10.3758/s13428-023-02194-2)
Supplement: Supplementary file 1 — (DOCX 2.03 MB) [file 13428_2023_2194_MOESM1_ESM.docx]

Online Supplement ESMira: A decentralized open-source application for collecting experience sampling data

David Lewetz^1^ & Stefan Stieger^1^

^1^Department of Psychology and Psychodynamics, Karl Landsteiner University of Health Sciences, Krems an der Donau, Austria

# Screenshots for the Main Features of ESMira

Complementary to the description of ESMira’s main features, this section provides additional screenshots. The screenshots are taken from the ESMira’s Admin Panel and the mobile app (either Android or iOS).

Figure S1.

ESMira’s Update Settings.


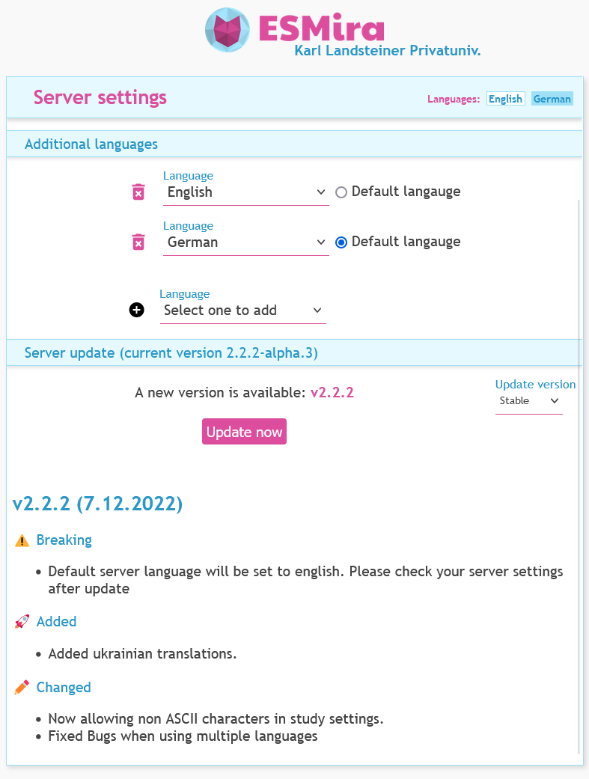


Figure S2.

Permission Management for a Specific User.


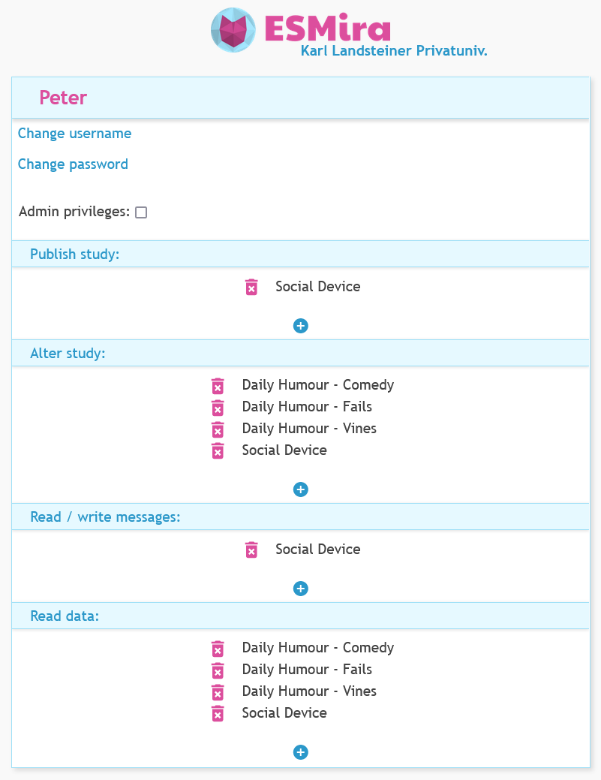


Figure S3.

A List of Available Data Files for a Study.


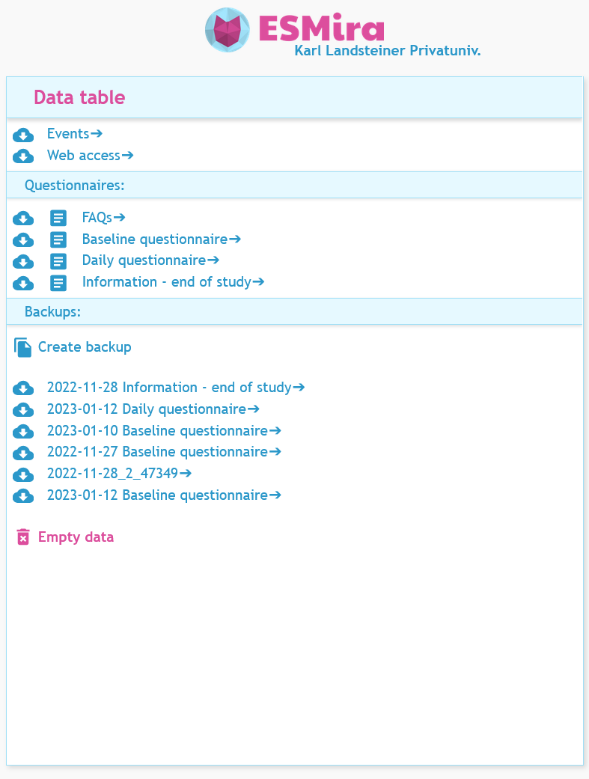


Figure S4.

The Online Data Viewer (Left) and a Filter Dialog of the Data Viewer (Right).


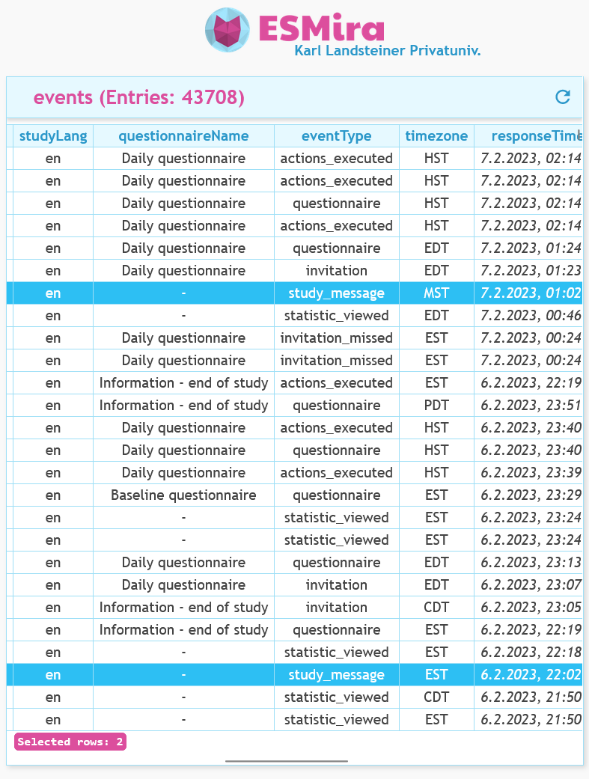

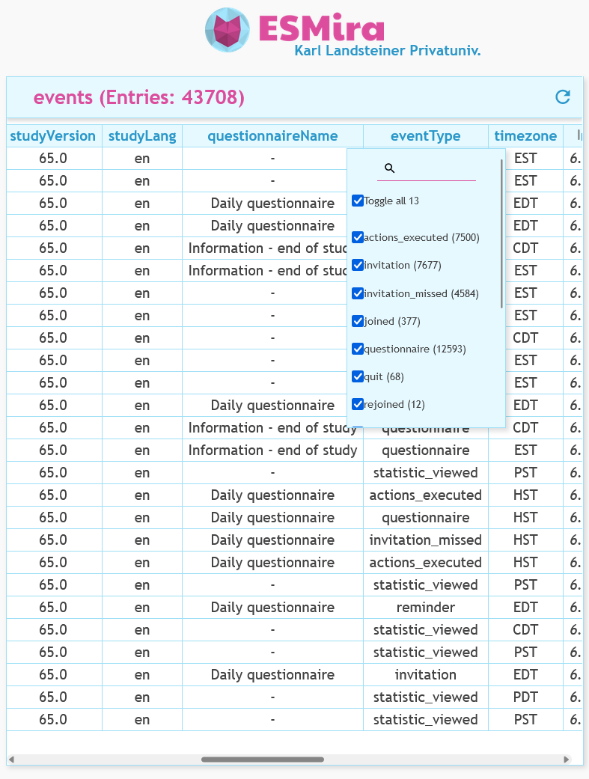


Figure S5.

The Summary Screen for a Specific Study.


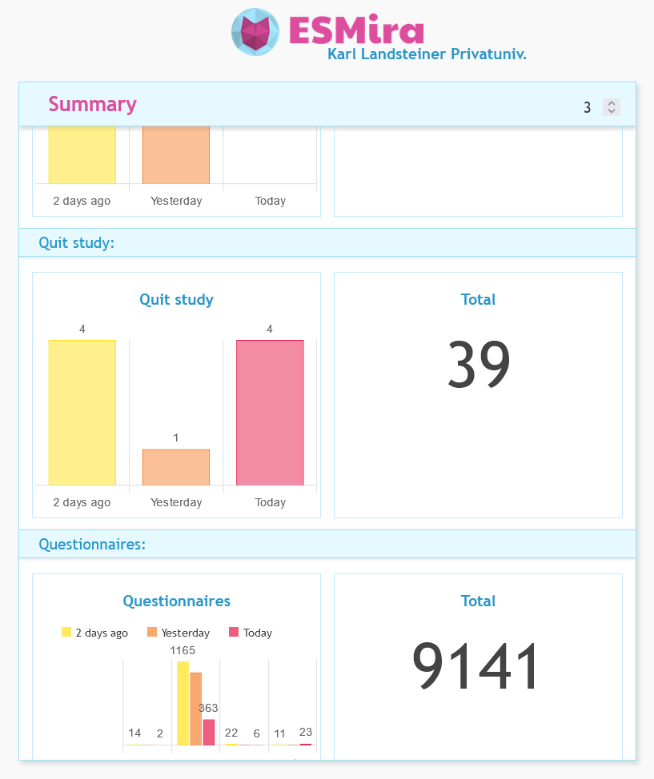


Figure S6.

Descriptive Statistics of a Particular Participant.


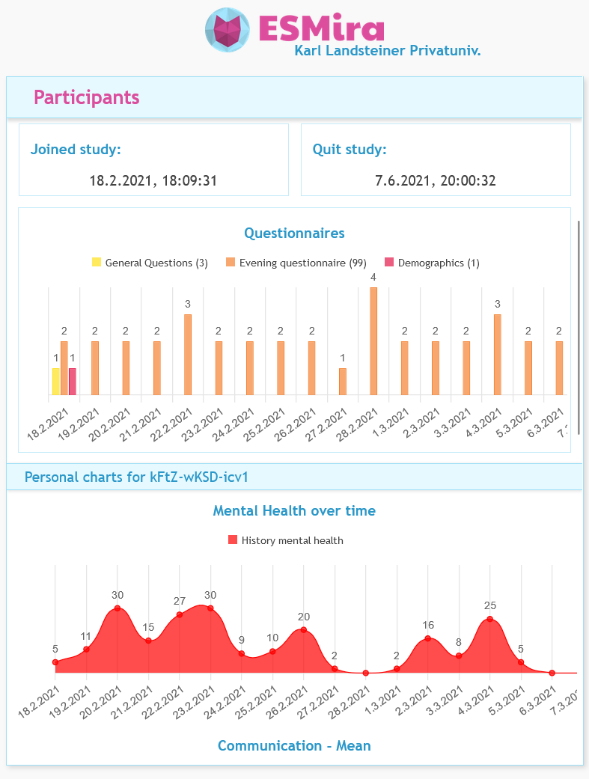


Figure S7.

Overall Statistics of a Server.


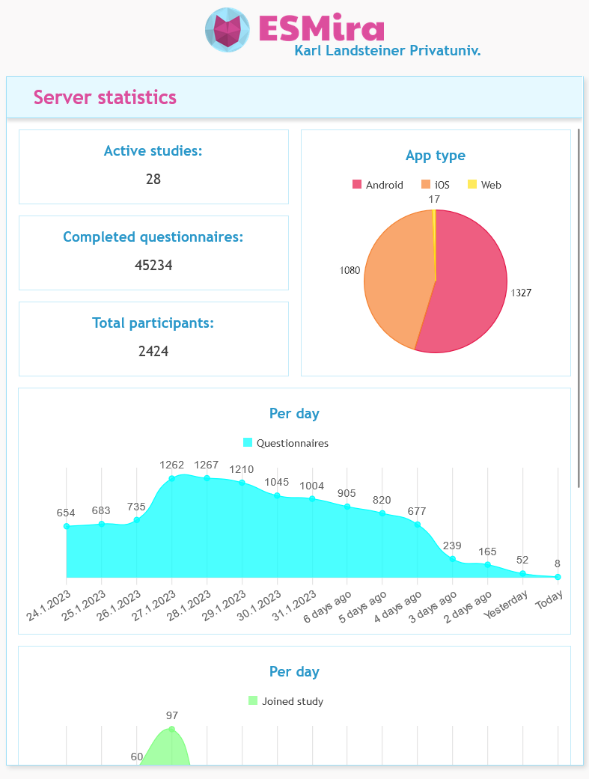


Figure S8.

An Example of the Automated Participation Instructions Screen.


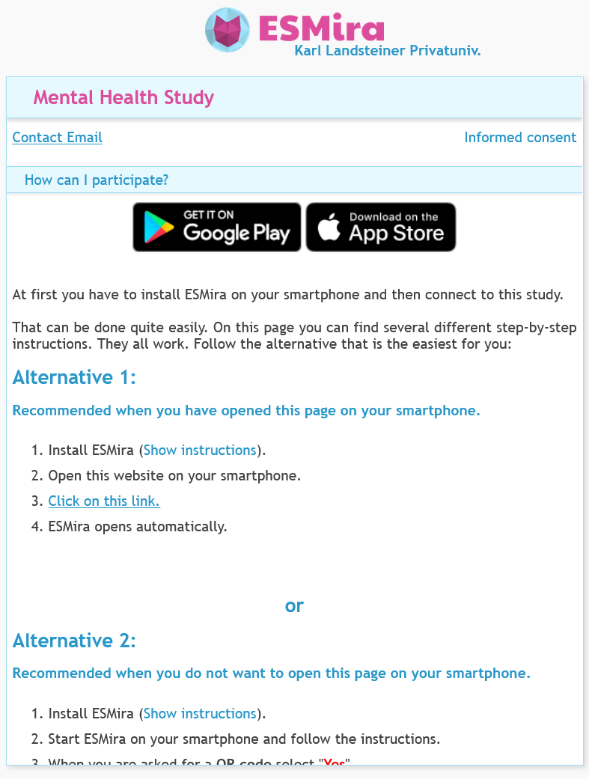

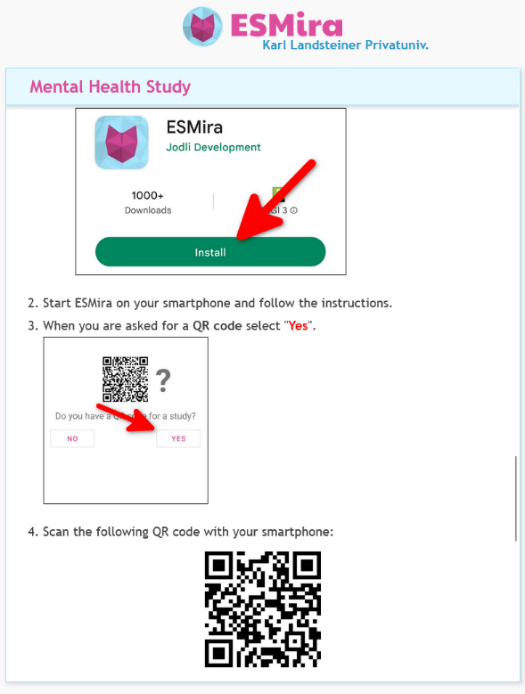


Figure S9.

An Example of the Web Interface for Questionnaires.


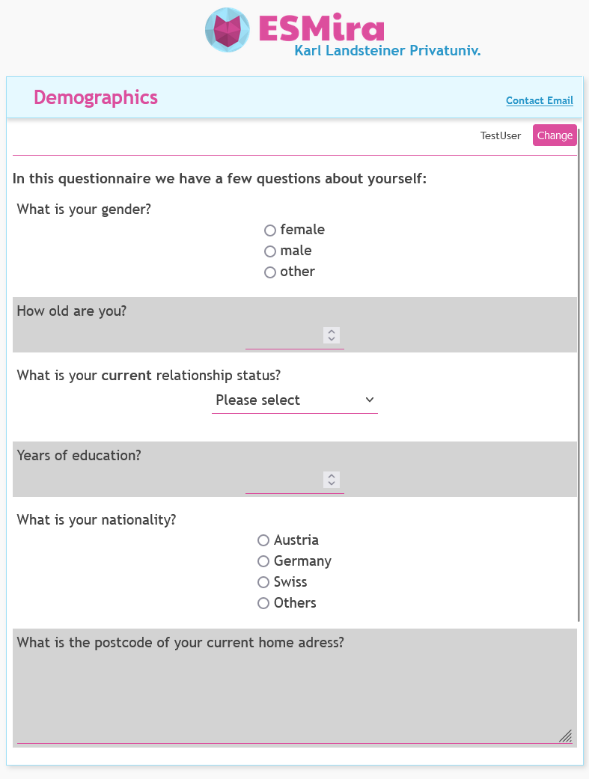


Figure S10.

Admin Interface for a Study with Multiple Languages and a Close up of a Language Selector for English and German.


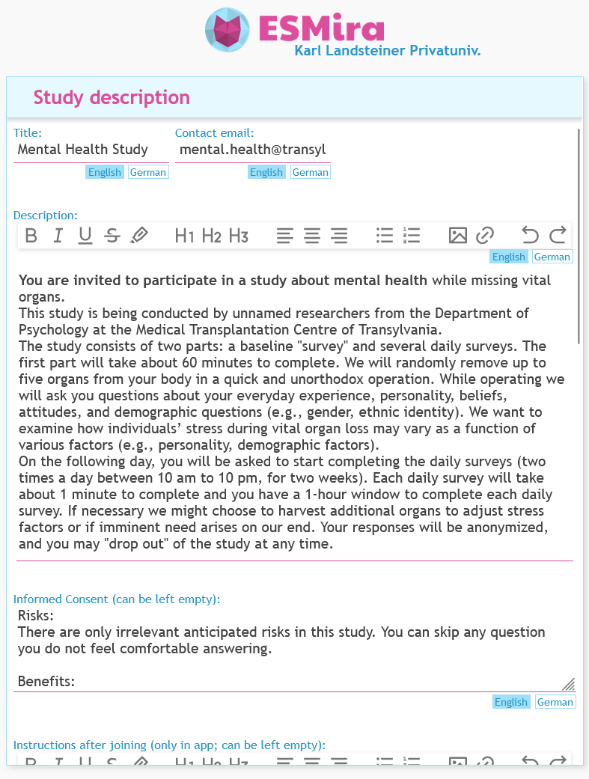

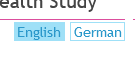


*Note*. Each text box has a language selector that can be used to change the currently selected language without having to reload the page.

Figure S11.

Settings for Random Groups and Study Languages.


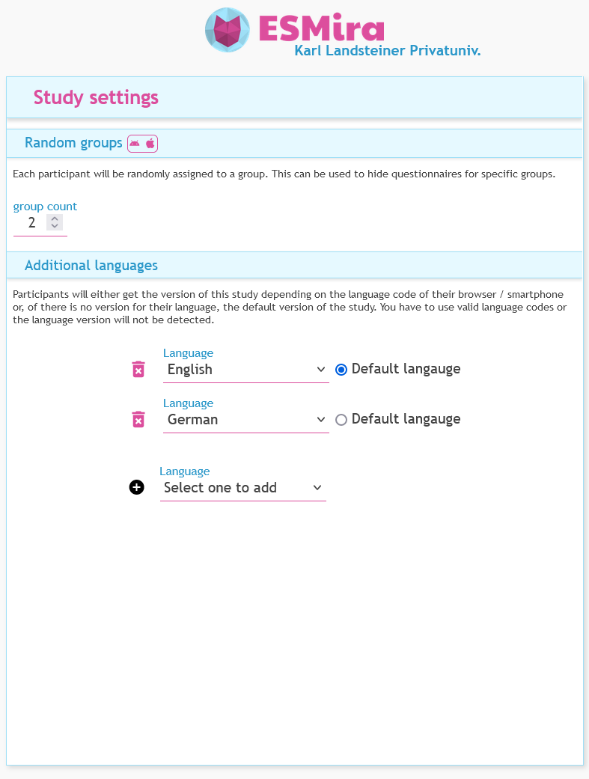


Figure S12.

Buttons for Saving Study (Left Tab) and Sending Changes to Existing Participants (Right Tab; i.e., Publish Changes Online).


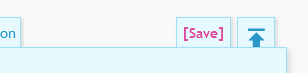


*Note*. When updating an existing study, changes will only be visible to new participants unless the “publish” button (arrow in right tab) is pressed. Then, apps from existing participants will automatically download the updated version of the respective study design file.

Figure S13.

Menu for Sum Scores Divided by Questionnaires.


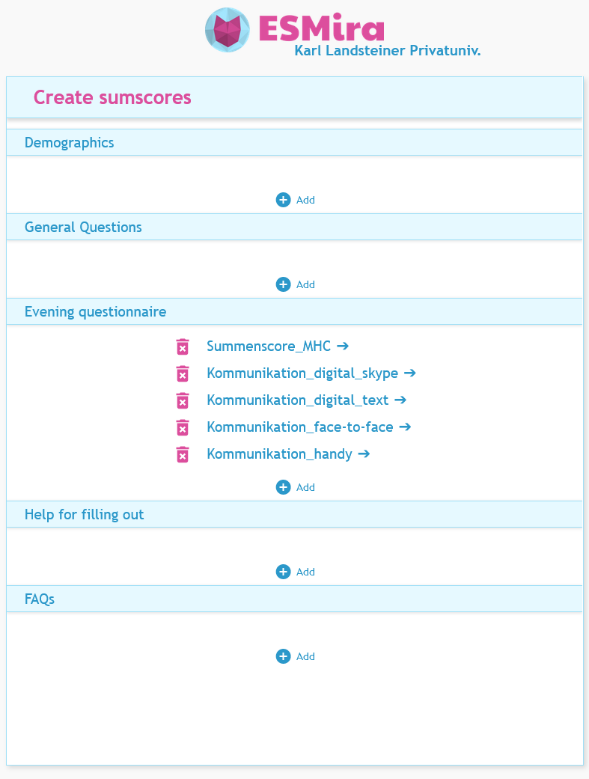

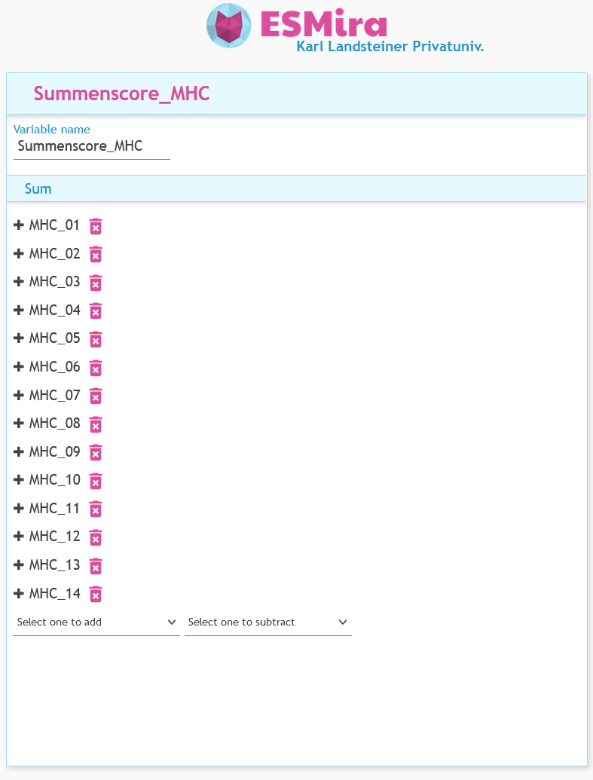


Figure S14.

View of the Study Design Source Code in Tree and JSON Format.


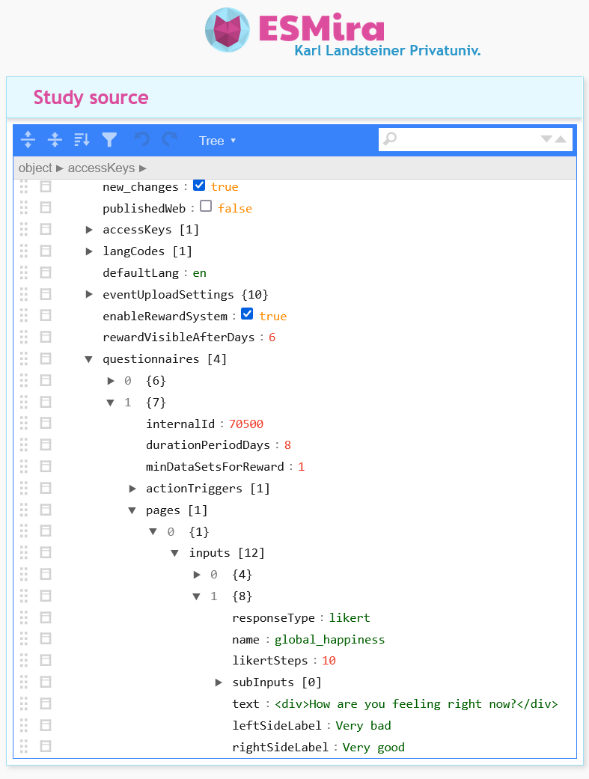


Figure S15.

Asking for a QR Code (Left), Instructions to Scan a QR Code (Middle) and Asking for Access Key (Right).


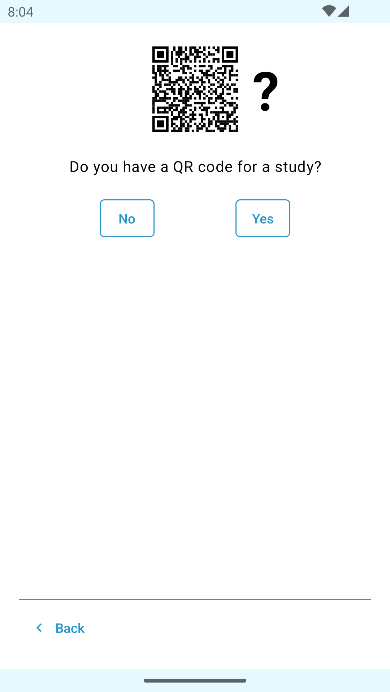

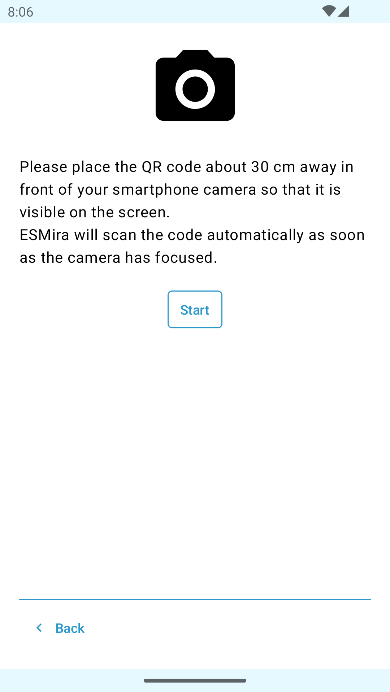

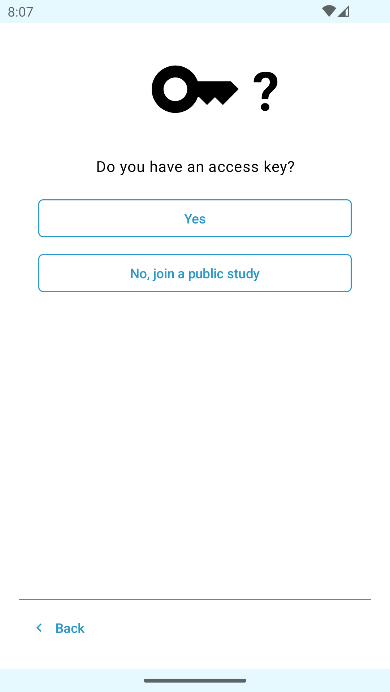


Figure S16.

Screen for Informed Consent (Left), Notifications (Middle) and App usage (Right).


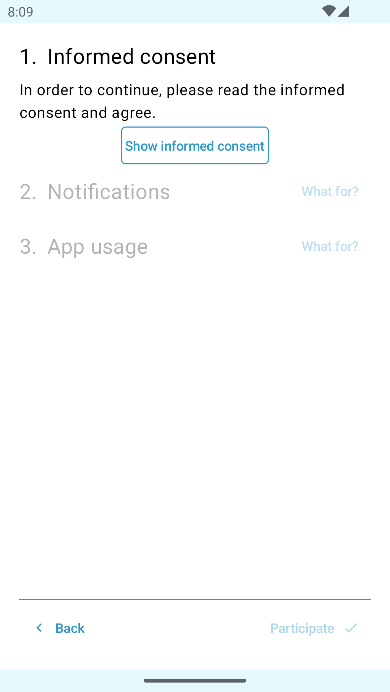

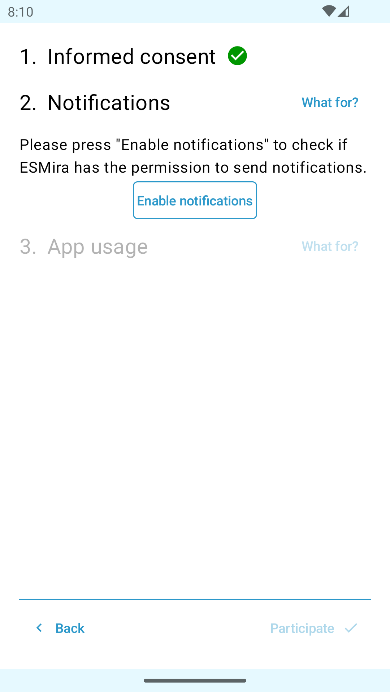

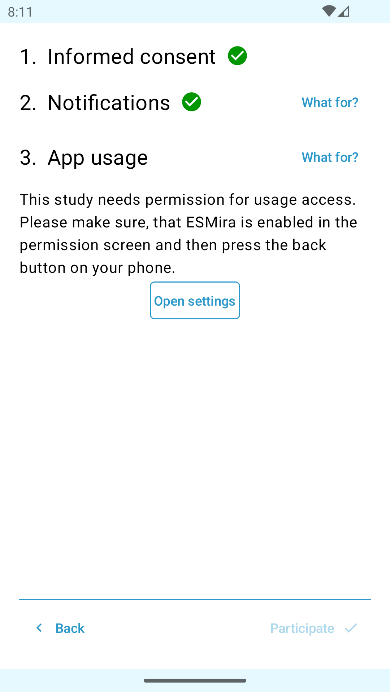


*Note*. When enrolling in a study, only the permissions that are required for that study are shown.

Figure S17.

Overview of all Triggers for a Study (Left) and Settings for One Specific Trigger (Right).


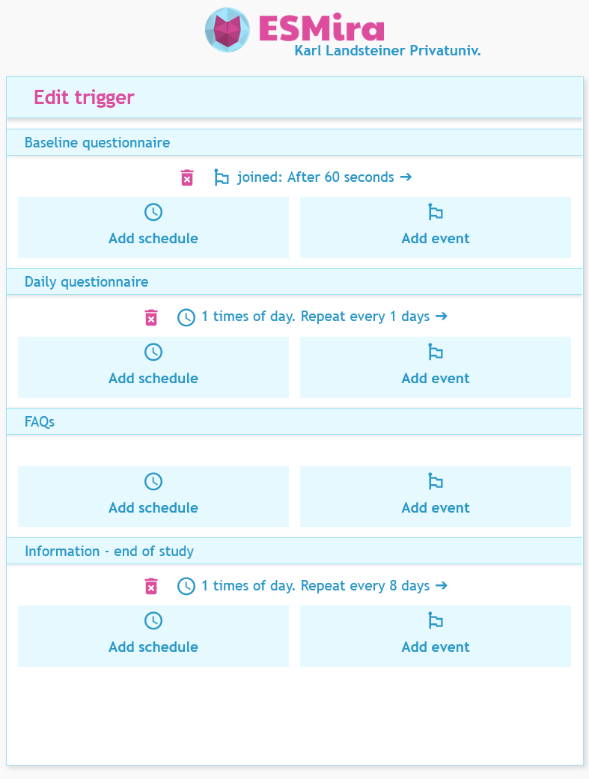

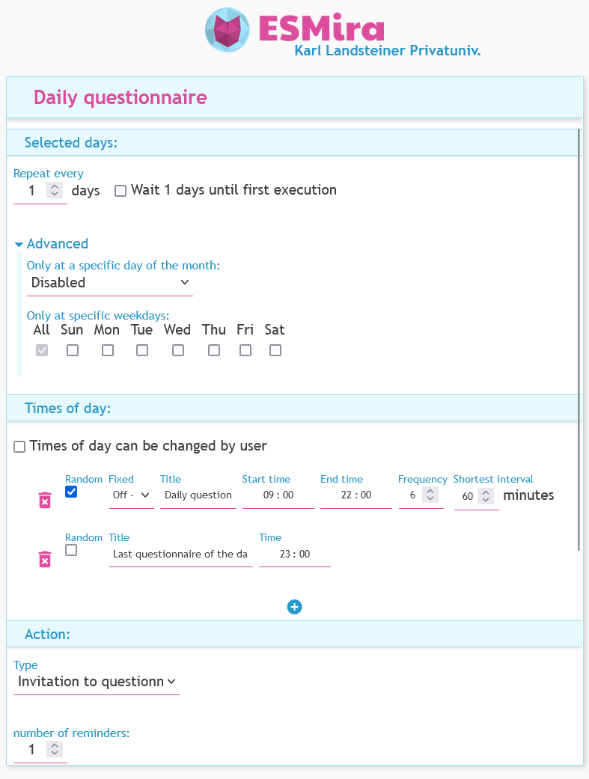


Figure S18.

An Example of an Anonymous Dialog in the Mobile App for Android (Left) and iOS (Right).


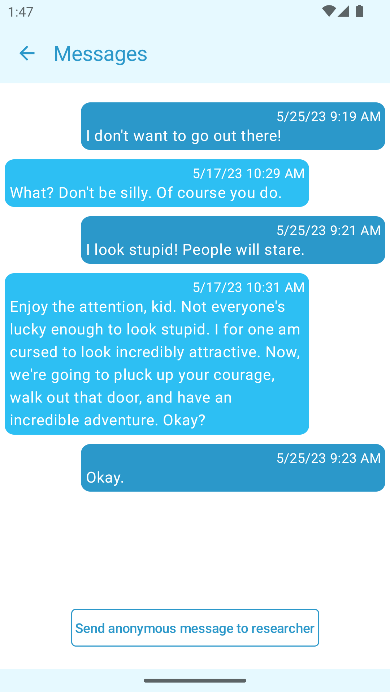

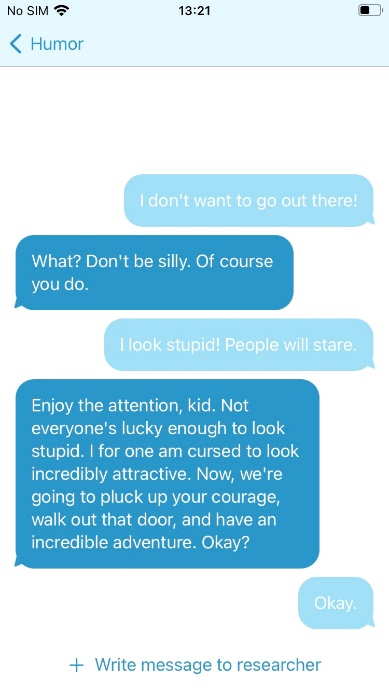


*Note*. This dialog is only fictional since, as far as we are aware, the characters from the movie *Jojo Rabbit* (Waititi, 2020) did not use ESMira to communicate.

Figure S19.

Several Charts from the Automated Feedback Option (Android Screenshots).


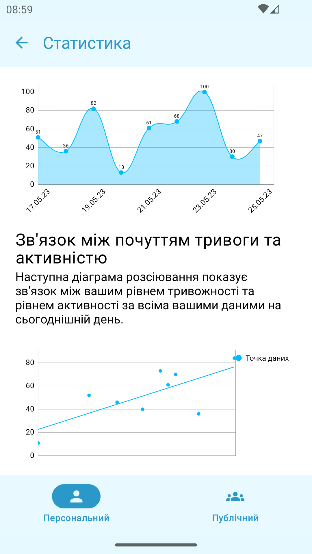

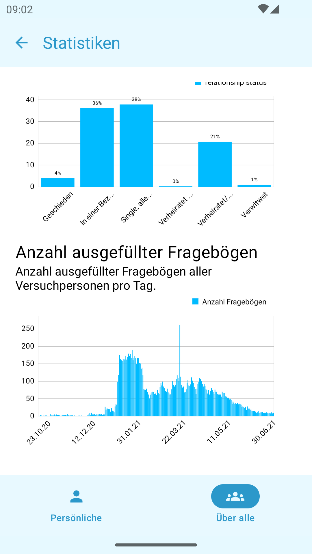

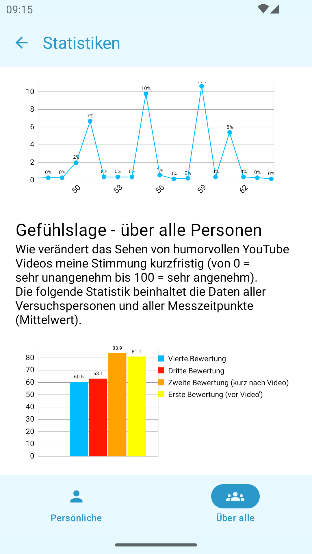

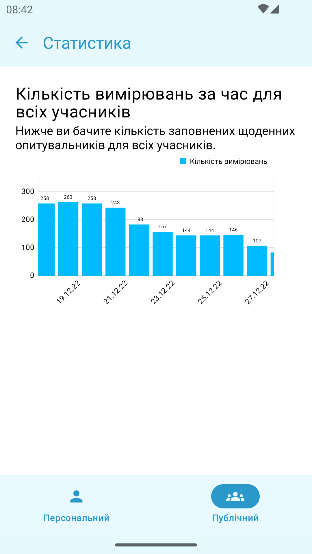


Figure S20.

Settings Screen for the Rewards of a Study (Left) and Screen for Validating Reward Codes (Right).


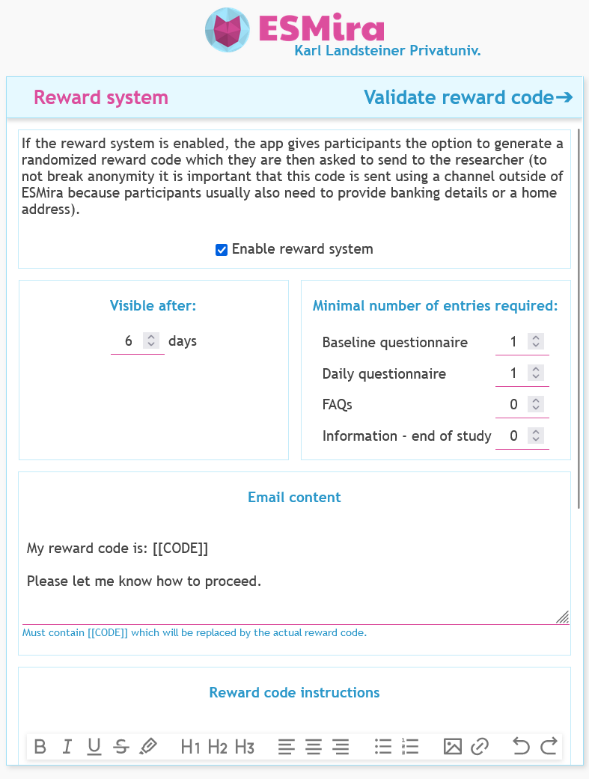

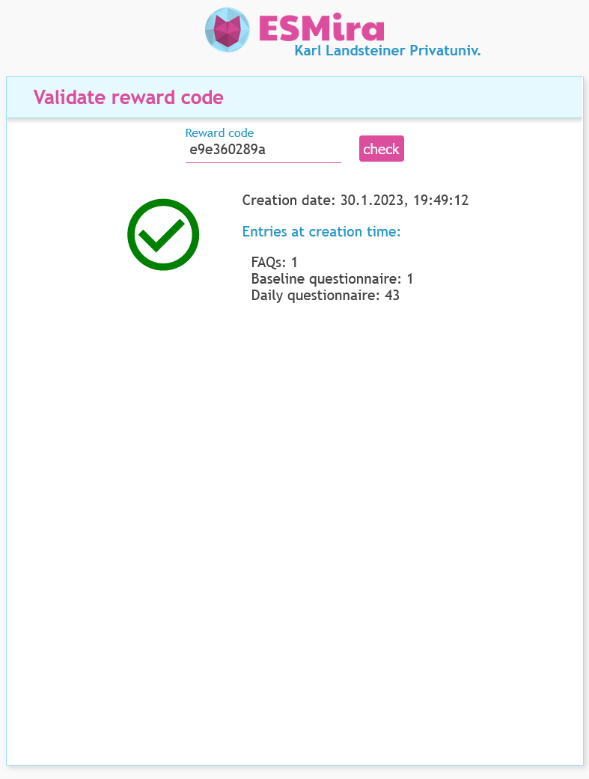


Figure S21.

The Rewards Screen in the Mobile App When Requirements are Fulfilled (Left) and not Fulfilled (Right).


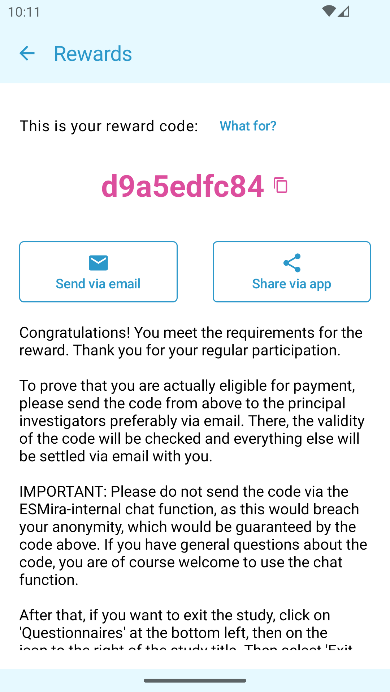

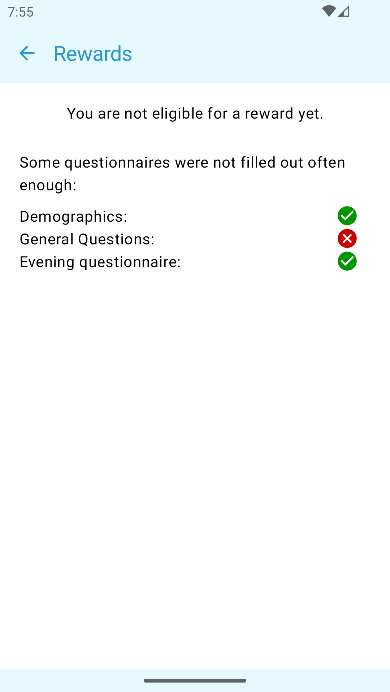


Figure S22.

Dialog to Change Schedule for all Pings That Have this Option Enabled for Android (Left) and iOS (Right).


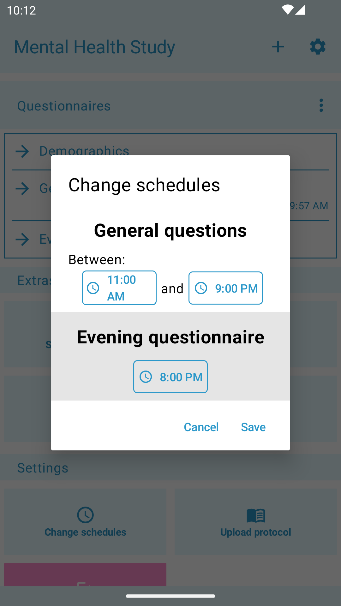

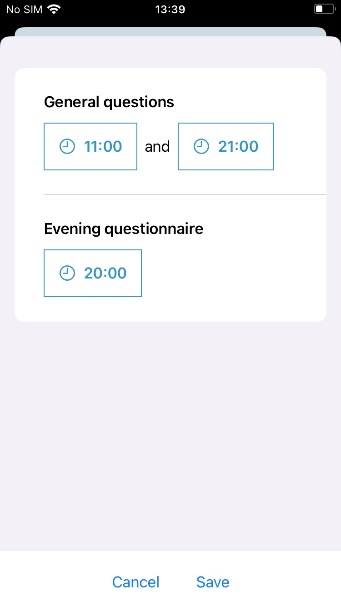


Figure S23.

Example of a Questionnaire with One Screen Time Item (Left) and a Questionnaire with Three App Usage Items (Right).


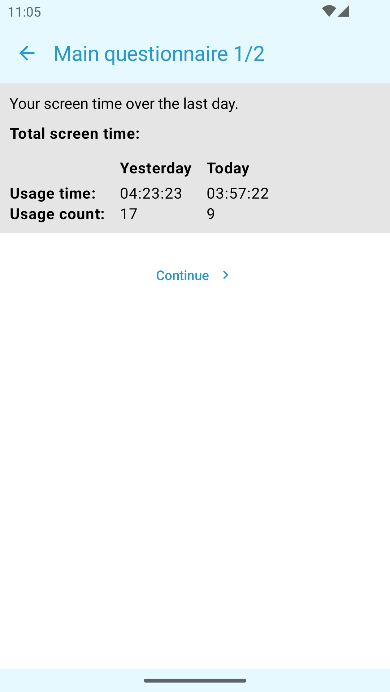

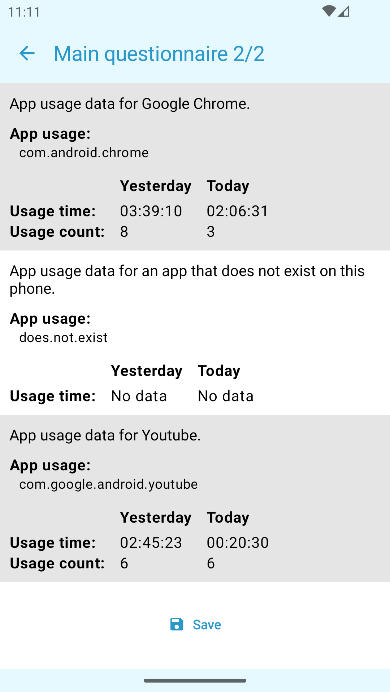


Figure S24.

Dialog That Automatically Opens on Android When ESMira Detects That Notifications Could Not be Issued Properly.


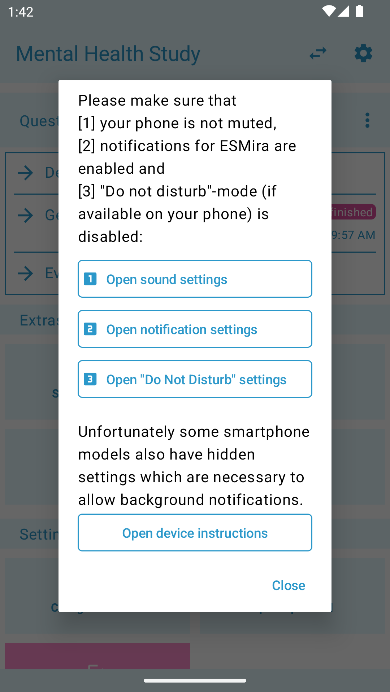


*Note*. The button labeled “Open device instructions” is only shown on specific Android models. It opens an additional dialog with instructions for the specific model of the user. Contents of the additional dialog are loaded over the internet from <https://dontkillmyapp.com>.

# Future Directions

ESMira has been in constant development since 2019. Over the years requirements have changed, solutions we liked at the time turned out to be insufficient and our list of features we would like to see in ESMira has grown steadily. Many entries on our “To Do list” will probably not be implemented in the foreseeable future, but there are some that we are prioritizing.

The design of the mobile app of ESMira has started out with just being a list of questionnaires that can be filled out (after a study was joined). And while, at its core, this is still its main function, it has become difficult to express all its different functionalities and options sufficiently with the original interface. One example was the original bottom menu which used to consist of only two entries (“questionnaires” and “settings”) and later also included “statistics”, “messages” and “rewards”. A growing issue with this menu was that we were simply running out of space (especially on small screen devices). Furthermore, we found that participants who are used to platforms where questionnaires can only be completed once (e.g., Prolific^[[1]](#footnote-2)^) were sometimes confused when the same questionnaire was shown multiple times or was still available after being filled out. For this reason, we just recently published a complete redesign of the user interface in an attempt to structure all functionalities of ESMira more clearly and to make it more coherent when and how often questionnaires are shown.

A similar issue still exists in the admin panel of the server. Over time, options and functionalities slowly grew in number and some areas are becoming overcrowded with options. We want to hide some of these options in “advanced” sections and create templates that bundle several, commonly used options that can be finetuned afterwards, if needed.

We also want to add more items that connect to smartphone sensors (e.g., GPS) which we had prepared from the beginning but so far had never used in any of our studies and therefore never implemented. Furthermore, adding Bluetooth connectivity is planned in the near future.

1. <https://www.prolific.co/> [↑](#footnote-ref-2)
